# Sustainable Bioethanol Production and Phenolic Compounds from Avocado Stone Biomass Based on Microwave Pretreatment

**DOI:** 10.3390/foods14183160

**Published:** 2025-09-10

**Authors:** Luis Carlos Morán-Alarcón, María del Mar Contreras, Juan Miguel Romero-García, Ángel Galán-Martín, Eulogio Castro

**Affiliations:** 1 Department of Chemical, Environmental and Materials Engineering, Universidad de Jaén, Campus Las Lagunillas, 23071 Jaén, Spain; lmoran@ujaen.es (L.C.M.-A.); jrgarcia@ujaen.es (J.M.R.-G.); galan@ujaen.es (Á.G.-M.); ecastro@ujaen.es (E.C.); 2 Institute of Biorefineries Research (I3B), Universidad de Jaén, 23071 Jaén, Spain

**Keywords:** avocado waste, bioethanol, biorefinery, hydroxycinnamic acids, diluted acid, lignin, microwaves

## Abstract

The transition towards sustainable biofuels requires innovative strategies to maximize the utilization of agroindustrial biomass. Accordingly, the aim of this study was to evaluate avocado stone biomass as a renewable substrate for producing glucose and bioethanol, and to characterize potential co-products from the pretreatment stream, including avocado phenolic compounds. It was found that the whole avocado stone and the seed contained 41.7% and 42.8% of starch, respectively, accounting for more than 78% of the glucans. Using microwave-diluted acid pretreatment and multi-response optimization, a direct conversion of ~90% of glucans to glucose was achieved from avocado stone biomass at 1% *w*/*v* sulfuric acid, 140 °C, and 5 min. It also enabled minimizing inhibitor presence and reducing energy requirements. Then, the glucose-rich hydrolyzate was efficiently fermented into bioethanol (~24 g/L in 12 h) using *Saccharomyces cerevisiae*, without needing detoxification or enzyme addition. Additionally, the process yielded a lignin-rich solid fraction with an enhanced higher heating value (about 1.4 times) compared to the original biomass and an extract with phenolic compounds like caffeoylquinic acids and hydroxytyrosol, which enhances the valorization potential of this underutilized biomass. The overall balance can be 240 kg/t of bioethanol, along with 2.5 kg/t of phenolic compounds and 376 kg/t of lignin-rich solid. Finally, this work exemplified, in a real-world scenario, how we can fully leverage these often-overlooked, non-edible sources of starch to achieve the green transition and circularity.

## 1. Introduction

Bioethanol offers a sustainable alternative to conventional fossil fuels, offering adequate thermal efficiency and closing the carbon cycle, as the released CO_2_ after combustion is again consumed by plants during photosynthesis to produce biomass. Therefore, its adoption contributes to the current efforts to decrease CO_2_-equivalent emissions and fossil fuel use [1,2], a priority, especially relevant in the transport sector, responsible for over 25% of global greenhouse gas (GHG) emissions [3]. Bioethanol can also be used as a key reactant intermediate to produce various chemicals [4], jet fuel [5] and polyethylene [1], and it has diverse applications as a green solvent, in the liquor industry, and as a sanitizing agent [6].

With the global demand for bioethanol expected to rise [6], developing sustainable and economically viable production methods from different biomass resources is crucial to meet future needs. Commonly, sugar- and starch-rich crops are used to produce bioethanol in first-generation (1G) biorefineries based on fermentation, e.g., sugar cane juice and corn grains [3]. However, this approach raises concerns about competition with their food application and uses [3,7], and the bioethanol profitability is limited by the high prices of the feedstock [4]. To address this issue, second-generation (2G) biorefineries offer a sustainable alternative by utilizing non-edible and inexpensive lignocellulose materials (e.g., forest and agroindustry wastes). However, despite their potential, 2G biorefineries still face challenges in the production steps [3]. For example, biomass pretreatment and enzymatic hydrolysis are crucial to achieving a high recovery of fermentable sugars; however, these stages increase the process cost [3,6]. For pretreatment, the application of dilute acids is well established on an industrial scale, but the effective management of this operation is necessary to minimize inhibitor generation [8]. Membrane–acid separation and acid reutilization [9] and the application of microwaves as a green heating technology [7] are among the strategies to overcome these limitations. The application of microwaves in the pretreatment facilitates the efficient and rapid conversion of biomass polysaccharides [7,10]. Moreover, to increase profitability and to support a more integrated and sustainable biorefinery model, recent trends focus on producing bioethanol alongside valuable chemicals from the sugar fraction [4] or co-producing other types of compounds from the extractive fraction (e.g., valuable phenolic compounds and carotenoids), pectin, and lignin [10,11,12]. Therefore, it is essential to evaluate the key factors of microwaves to achieve energy-efficient pretreatment of agroindustrial biomass, resulting in high bioethanol performance and facilitating the production of valuable co-products. This is because most studies have employed conventional heating methods in biorefinery proposals.

One promising feedstock for 2G biorefineries is avocado processing waste, a growing agroindustrial waste worldwide due to the increasing demand for guacamole, frozen avocados, avocado oil, sauces, snacks, and cosmetics. This industry discards the avocado peel and stone as waste, which may represent more than 24% *w*/*w* of the fruit [13]. For example, in Mexico, the world’s leading avocado producer, 19% of the production is destined for industrial processing [14], generating substantial waste streams located in the industrial plants. However, previous studies on avocado stone waste composition reveal large variations [13,15,16,17], highlighting the need for standardized methodologies to define the chemical composition of the stone parts more accurately and more clearly. Moreover, while a stand-alone process has been applied to obtain bioethanol from avocado waste [17], integrated biorefining and cascading approaches remain largely unexplored at the laboratory scale to support their application at larger scales.

The research hypothesis was to find out if microwave-assisted diluted acid pretreatment of avocado stone biomass enables the efficient production of bioethanol within a biorefinery scheme. Hence, the objective of this work was to develop a novel, integrated valorization strategy for avocado stone biomass by optimizing microwave-assisted diluted acid pretreatment to maximize glucose release, produce bioethanol, and characterize potential co-products of the process. The approach began with a comprehensive chemical characterization of the avocado stone fractions (seed coat, seed, and stone) to establish a clear understanding of its composition. The process was then evaluated and optimized through microwave-assisted diluted acid to maximize the production of rich-glucose hydrolyzates from avocado stone biomass while minimizing inhibitory load, energy consumption, and severity aspects by multi-response optimization. The resulting glucose-rich hydrolyzates were subsequently and directly fermented into bioethanol using *Saccharomyces cerevisiae*, thereby eliminating the need for detoxification and enhancing process efficiency and sustainability. Additionally, the study explored the characterization of potential co-products, including avocado phenolic compounds and the pretreated solids generated during the pretreatment steps, which could further improve the economic and environmental viability of the approach. For example, avocado phenolic compounds have potential for developing natural preservatives and functional ingredients [10], thereby increasing the value of agroindustrial by-products while satisfying the rising demand for natural bioactive ingredients. Overall, this is the first time that avocado stone biomass has been valorized integrally using a simplified and efficient conversion process based on dilute acid and microwaves.

## 2. Materials and Methods

### 2.1. Raw Biomass Conditioning

**Figure 1:**
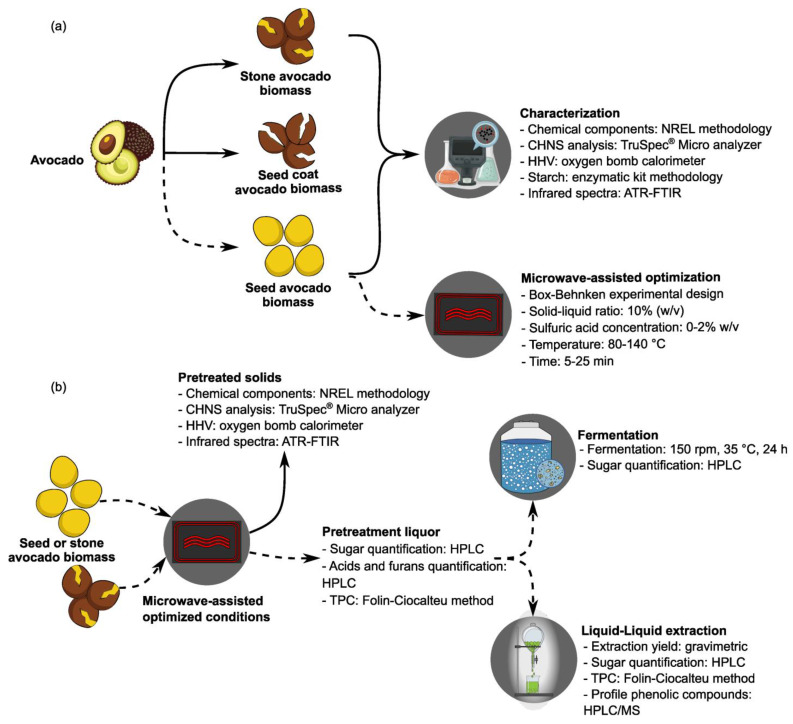
Scheme of the procedures and analytical methods applied: (**a**) Characterization of the samples (avocado stone, coat, and seed) and optimization of the microwave-dilute acid pretreatment for the avocado seed. (**b**) Implementation of the pretreatment under optimal conditions (1% *w*/*v* sulfuric acid, 140 °C for 5 min) on avocado seed and stone. Abbreviations: ATR-FTIR, Attenuated Total Reflectance-Fourier Transform Infrared Spectroscopy; CHNS, carbon, hydrogen, nitrogen, and sulfur; HHV, high heating value; HPLC, high-performance liquid chromatography; MS, mass spectrometry; NREL, National Renewable Energy Laboratory; TPC, total phenolic content.

The avocados (cultivar ‘Hass’) were bought at a nearby supermarket (Jaén, Andalusia, Spain). The fruit was cleaned, and its components were manually separated and weighed fresh using a precision balance. The separated components included peel (or epicarp), pulp (flesh or mesocarp), and stone. The stone was further fractionated into the seed coat (or endocarp) and the seed (Supplementary Materials Figure S1). All fractions were then dried at 45 °C until their moisture level was below 10% with the following final values: 7.51 ± 0.05% (seed coat), 5.07 ± 0.32% (seed), and 5.27 ± 0.01% (stone). Once dried, the samples were subsequently ground using a ZM 200 Model Ultra-Centrifugal Mill with a 1 mm sieve (Retsch GmbH, Haan, Germany). The resulting samples were stored at room temperature until further analysis, as described in the sections below and schematized in Figure 1.

### 2.2. Reagents

The following reagents were of analytical grade. They were supplied by Panreac Applichem ITW Reagents (Barcelona, Spain): sulfuric acid 72%, hexane, glucose, xylose, galactose, arabinose, mannose, ethyl acetate, and sodium hydroxide. The reagents from Sigma-Aldrich (St. Louis, MO, USA) were: gallic acid, Folin & Ciocalteu’s phenol reagent, the studied organic acids, furfural, and hydroxymethylfurfural (HMF). Other reagents were: sodium carbonate (Honeywell Fluka, Seelze, Germany), sulfuric acid 96% (J.T. Baker, Avantor, Radnor, PA, USA), acetonitrile, and methanol ≥ 99.8% type (VWR Chemical, Avantor). Ultrapure water was produced using a Milli-Q system (Millipore, Bedford, MA, USA).

### 2.3. Chemical Characterization of the Raw Biomass and Pretreated Solids

#### 2.3.1. Chemical Composition

The moisture and ash contents were gravimetrically determined after drying at 105 °C in a UF110 oven (Memmert GmbH, Schwabach, Germany), using a muffle furnace (Nabertherm, Lilienthal, Germany) set at 575 °C for the raw and pretreated solids.

The extractives of the raw biomasses were quantified using sequential Soxhlet extraction with water and ethanol (24 h for each step), followed by drying and weighing the extracted material. The remaining extracted solids and the pretreated solids were subjected to a two-step acid-hydrolysis to determine the polymeric sugars and the lignin content following the standardized National Renewable Energy Laboratory (NREL) methodology [18]. The sugars (glucose, xylose, galactose, arabinose, and mannose) of the aqueous extracts (Soxhlet) and the acid hydrolyzates obtained by the NREL procedure were quantified using high-performance liquid chromatography (HPLC) presenting a refractive index detector (RID) (2695 Waters; Milford, MA, USA), according to García-Vargas et al. [19]. The samples were injected (20 μL) onto a column, which was CARBOSepCHO-782 lead type, 300 mm × 7.8 mm (Transgenomic Inc., Omaha, NE, USA), maintained at 70 °C, and with Milli-Q water flowing at 0.6 mL/min.

Moreover, the lipid content of the raw biomasses was also determined by performing Soxhlet extraction with hexane. The total starch content was measured using an enzymatic kit methodology (100 A) (Megazyme, Wicklow, Ireland), as per the manufacturer’s instructions. Briefly, it is based on the use of α-amylase, which converts starch to maltodextrin, and then amyl glucosidases hydrolyze the maltodextrin to glucose, which is spectrophotometrically quantified (Biochrom Libra, Cambridge, UK).

All results were estimated as g/100 g biomass on a dry basis, except moisture reported on a fresh basis.

#### 2.3.2. CHNS and Energetic Content Determination

The carbon (C), hydrogen (H), nitrogen (N), and sulfur (S) contents (% *w*/*w*) of the samples (about 2 mg) were measured using the TruSpec Micro device (Leco, St. Joseph, MI, USA). Briefly, it is based on the combustion of the samples and an analysis of the produced gases by infrared absorption (CO_2_, H_2_O, and SO_2_) and thermal conductivity detection (N_2_) [20].

The higher heating value (HHV) was estimated using the C content (% *w*/*w*); Equation (1), which is appropriate for biomass, including that with a high lignin content [21]:

(1) $$HHV\;\left(\frac{kJ}{g}\right)\;=\;0.4373\;\times \;C\;-\;1.6701$$

The *HHV* was also experimentally measured using an oxygen bomb calorimeter (model 6050) from Parr Instrument Company (Moline, IL, USA) after pelleting the samples with a CARVER laboratory press (model C) (Wabash, IN, USA).

#### 2.3.3. Infrared Spectroscopy

Moreover, attenuated total reflectance (ATR)-Fourier transform infrared (FTIR) spectroscopy was performed using a Bruker Vertex 70 device, equipped with ATR platinum and a DLaTGS/KBr detector (Bruker, Bremen, Germany). Spectra were measured as follows: 400–4000 cm^−1^, 4 cm^−1^ resolution, and 32 scans per measurement. They were obtained and visualized with OPUS 8.7.31 software from Bruker.

### 2.4. Microwave-Assisted Diluted Acid Pretreatment and Design

The pretreatment of the avocado stone and seed was performed in closed reactors within a multimode microwave system using pressurized Teflon vessels (Milestone Srl, Sorisole, Italy).

First, to evaluate the operational factors on the microwave pretreatment, a Box–Behnken experimental design (BBD) was applied to the seed fraction, consisting of 17 randomized experiments with five central points. The experimental design was analyzed as described in Section 2.9. The following parameters were fixed: agitation at 300 rpm (70%), and the sample solid loading was 10% (*w*/*v*). The independent variables included sulfuric acid concentration (0, 1, and 2% *w*/*v*), temperature (80, 110, and 140 °C), and time (5, 15, and 25 min). The samples were centrifuged (4000 rpm and 10 min) (Herolab HiGen T model, Berlin, Germany), and the resulting supernatants (pretreatment liquors) and solids (pretreated solids) were subsequently separated (Figure 1). A portion of each pretreatment liquor was filtered using syringe filters (0.45 µm pore size, nylon) (Sinerlab) (Madrid, Spain) for the quantification of sugars (including glucose) and inhibitors (see Section 2.5 and Section 2.8 below). In addition, the liquors were subjected to a second hydrolysis step using 96% diluted sulfuric acid at 120 °C for 30 min in an autoclave (Raypa, Barcelona, Spain). This step allows for estimating the glucose in the liquors, including monomeric and oligomeric forms, when glucans were not fully converted into glucose during the initial pretreatment.

A current consumption meter (Gifort, China) was used to monitor the energy consumption (kWh). Moreover, for each experiment, the combined severity factor (CSF) was estimated using the following Equation (2), according to the methodology described in a previous study [22] for acid-catalyzed pretreatments:

(2) $$CSF\;=log\left[t\times exp\left(\frac{T-100}{14.75}\right)\right]-pH$$

The CSF considers the combined effects of the residence time (t, min), residence temperature (T, °C), and pH of the initial sulfuric acid solution applied for the pretreatments.

Second, the dilute acid pretreatment assisted by microwaves was evaluated and optimized using the response surface methodology (RSM) for the seed, and the optimal conditions were applied to hydrolyze the seed and stone (seed plus coat) biomass (see Section 2.9).

### 2.5. Determination of Sugars and Inhibitors in the Pretreatment Liquors

Glucose, arabinose, and xylose, acetic acid, formic acid, levulinic acid, and galacturonic acid, and furans (furfural and HMF) were quantified (g/L) by 1260 HPLC-RID (Agilent Technologies, Santa Clara, CA, USA) with an ICSep ICE-COREGEL 87H3 column from Transgenomic Inc., set at 65 °C, according to a previous study [19]. Isocratic elution was performed using 5 mM sulfuric acid at 0.6 mL/min. Before analysis, all samples (standards, pretreatment, and fermentation liquors) were filtered (0.45 µm nylon syringe filters (Sinerlab) (see Section 2.4), and 20 µL was injected. The studied compounds were quantified using their respective external standards; however, xylose, galactose, and mannose were collectively quantified as xylose because they cannot be separated under these analytical conditions.

### 2.6. Fermentation

A portion of each pretreatment liquor (optimized conditions) from the stone and seed samples was neutralized at pH 5 with sodium hydroxide and subjected to fermentation with *Saccharomyces cerevisiae* (Ethanol Red^®^, Fermentis, Marquette-lez-Lille, France) (Figure 1). The inoculum culture and fermentation were carried out in 100 mL Erlenmeyer flasks at 150 rpm and 35 °C for 24 h, in triplicate, according to Romero-García et al. [23]. Samples were collected at 6, 12, and 24 h, centrifuged (Herolab Microgen 16), filtered, and analyzed for glucose and ethanol concentrations using HPLC-RID (as described in Section 2.5). The ethanol production yield obtained was calculated according to Equations (3) and (4).

(3) $$\%\;ethanol\;yield\;=\;\frac{experimental\;ethanol\;(\frac{g}{L})}{potential\;ethanol\;(\frac{g}{L})}\times 100$$

(4) $$Potential\;ethanol\;=\;glucose\;\left(\frac{g}{L}\right)\times 0.51$$

### 2.7. Liquid–Liquid Extraction of Pretreated Liquors

To assess the potential for recovering phenolic compounds, a separate portion of the pretreatment liquors, which were obtained under optimal conditions from the stone and seed, was extracted with ethyl acetate, according to a previous study [24]. The liquid–liquid extraction was performed at room temperature with a 1:3 (*v*/*v*) liquor-to-solvent ratio at 250 rpm for 15 min. The organic phase was subsequently evaporated, and the remaining solids were dissolved with distilled water to the initial volume. Before analysis, the samples were filtered (Section 2.4).

### 2.8. Characterization of Phenolic Compounds

The total phenol content (TPC) of the Soxhlet extracts, pretreatment liquors, and extracts recovered with ethyl acetate was determined by the colorimetric method of Folin–Ciocalteu at 655 nm Bio-Rad iMark reader (Hercules, CA, USA), following a previously detailed protocol, in triplicate [19]. The data were obtained in terms of g gallic acid equivalents (GAE)/L or normalized to biomass as mg GAE/g biomass.

Additionally, the latter extracts were analyzed using an Agilent 1200 HPLC device and quadrupole-time-of-flight (QTOF) mass spectrometry (MS) (Agilent 6530B) through electrospray. The acquisition mode was performed using both negative and positive ionization modes. The analytical methodology was similar to the previously described approach [25], but with an injection volume of 8 μL. For compound characterization, the molecular formula and fragmentation patterns of detected phenolic compounds were compared with previous studies on avocado seed and seed coat [26,27,28] and with the MassBank database, when the MS/MS data were available [29].

### 2.9. Statistical Analysis

For process optimization using RSM, optimal conditions for each model were identified based on the results of the seed fraction experiments. The statistical treatment of the BBD was evaluated for significance (*p* < 0.05) using Design-Expert^®^ V8.0.7.1 (Stat-Ease Inc., Minneapolis, MN, USA). The software provided analysis of variance (ANOVA), regression model equations, coefficient of determination (R^2^), lack-of-fit (*p*-value), and coefficient of variance (CV) to assess model accuracy. For each response variable, a second-degree polynomial equation was obtained Equation (5):

(5) $$y\;=\beta_{0}+\sum_{i=1}^{3}\beta_{i}x_{i}+\sum \sum_{i<j=1}^{3}\beta_{ij}x_{i}x_{j}+\sum_{i=1}^{3}\beta_{ii}x_{i}^{2}\;$$

In this equation, y is the response variable, $\beta_{0}$, $\beta_{i}$, $\beta_{ij}$, $\beta_{ii}$ are the regression coefficients obtained by the least squares method, and $x_{i}$, $x_{j}$, are the corresponding factors. The models were refitted with significant factors (*p* < 0.10), and the final statistical parameters were reported accordingly. Equations were obtained for: total glucose (glucose and glucose oligomers), glucose in monomeric form, total sugars, the combined content of acids and furans, TPC, and the consumed energy by microwaves.

Then, multiple-response optimization (desirability function) was applied with the aforementioned software, as in previous work [20], to maximize the production of total glucose, glucose in monomeric form, and total sugars while simultaneously minimizing the inhibitors (acids and furans) and the consumed energy. The optimal conditions were replicated experimentally three times on the seed to validate the optimization results, and the relative error for each response was calculated, considering the theoretical predictions provided by the software. The optimized conditions were reproduced using not only the seed part but also the entire stone (seed plus coat) for a comparative analysis of fermentation potential, phenolic content, and derived pretreated solids.

In addition, for the characterization data, the F-test was used to determine whether the variances of two samples are equal, and based on this result, the appropriate *t*-test was applied to evaluate whether their means differ using Microsoft Excel (Microsoft Office Professional Plus 2019) (Redmond, WA, USA).

## 3. Results and Discussion

### 3.1. Raw Biomass Characterization

On a fresh basis, the avocado fruit composition was 13.7 ± 2.2% peel, 68.8 ± 2.7% pulp, 0.6 ± 0.1% seed coat, and 16.9 ± 0.5% seed, indicating the stone (seed coat plus seed) accounts for approximately 17.5% of the fruit (Table 1; Supplementary Materials Figure S1). In total, the sum of potential residues accounts for approximately 31% of the fruit, although this value and the moisture content can vary slightly depending on the origin, growing conditions, cultivar, and avocado class [13,30]. For example, the values of avocado peel and stone have been reported to be between 24% and 49% *w*/*w* [30].

The current study reveals that the chemical composition of the avocado stone parts shows some differences (Table 1). For example, the seed coat contained higher levels of aqueous and hexanic extractives, acid-insoluble lignin, protein, and ash compared to the seed (*p*-value < 0.05). Conversely, the seed had greater amounts of ethanolic extractives and glucans (*p*-value < 0.05). Despite these differences, the avocado stone and seed here had a similar chemical composition, as the seed coat comprised only a small part of the stone, i.e., 3.4% and 4.5% on a fresh and dry basis, respectively. The chemical characterization of the avocado seed/stone has been previously studied; however, notable variability has been described across the literature, especially for the glucans, hemicellulose, and lignin content [10,13,15,16,17]. Some reported values for the avocado stone were 6.5–40.9%, 3.0–47.9%, and 1.8–15.8%, respectively [10,13,15,16,17]. Other studies found glucan contents of 53.6% and 57.3% in the seed, while the hemicellulose and lignin contents remained within the previous range [10,13,15,16,17]. Additionally, there is currently no information available regarding the composition of the seed coat. Thus, the provided information can assist in future studies on the utilization of avocado stone or its parts, following common standardized methodologies.

Notably, the glucans in the seed and stone were similar (*p*-value > 0.05) and mainly in the form of starch, with a content of approximately 42%. The total glucan content measured in this study (52.17% and 53.30%, respectively) falls within the range of previous studies on the cultivar ‘Hass’ (about 30–57%) [10,13]. However, the starch content was lower than that previously reported on this cultivar (64.9%) [17]. Other studies had reported starch levels of 66.3% [16] and 24.6% [31], depending on the origin, growing conditions, cultivar, and methodology, among other factors [13,30]. Additionally, the aqueous extractive fraction from both the seed and stone contained free glucose as the predominant sugar, contributing approximately 3% more to the glucose potential of these biomasses, as shown in Table 1. This finding reinforces the potential of avocado seed and stone as nonconventional starch sources, comparable to other fruit seeds like litchi and mango seed, which are rich in starch (>53%) [32]. Biorefineries that utilize this type of waste as feedstock can take advantage of starch-rich resources while avoiding competition for food and ensuring food security, compared to 1G biorefineries.

Regarding the ultimate analysis, the carbon content was slightly higher in the seed coat than in the seed (*p*-value < 0.05), which corresponded to an increased HHV, probably explained by its higher lignin content (Table 1). The carbon content and the estimated HHVs for the avocado stone were similar to those of the seed (*p*-value > 0.05), being within or close to previously reported ranges (42.1–48.0% and 15.2–19.2 kJ/g, respectively) [13].

### 3.2. BBD of the Pretreatment: Analysis of the Pretreatment Liquors

Given the high starch content (42.78%) in avocado seeds, this study focused on optimizing pretreatment parameters to efficiently release glucose and minimize barriers to starch conversion. Avocado seed was subjected to microwave-assisted diluted acid pretreatment following a BBD to evaluate key factors and optimize the production of glucose for bioethanol production. Table 2 shows the experimental conditions applied and the results obtained at 10% *w*/*v* solid loading. The total glucose concentration (including glucose and glucooligosaccharides) varied between 3.26 (experiment 8: 80 °C, 15 min, 0% acid) and 59.48 g/L (experiment 5: 140 °C, 5 min, 1% *w*/*v* acid). Under the latter conditions, nearly all the glucose was recovered, along with other sugars (14.4 g/L) in the pretreatment liquor. This indicates that starch was efficiently hydrolyzed into glucose under moderate sulfuric acid concentration at 140 °C.

Compared to previous studies, the results demonstrate good efficiency and yield, as glucose was directly recovered in a single microwave-based pretreatment step, without the need for enzymatic hydrolysis. For example, in a previous study, microwave-assisted autohydrolysis was applied at 220 °C for 5 min, yielding 37.80 g/L glucooligosaccharides from avocado seed. However, this process required an additional two-step enzymatic hydrolysis with Termamyl^®^ SC 4X (3 h, 90 °C) and Saczyme^®^ Yield (15 h, 50 °C) to convert the glucooligosaccharides to glucose [10]. Similarly, another study employed conventional heating at 87 °C for 12 h, combined with diluted sulfuric acid (2% *w*/*w*) at 15% *w*/*v* of avocado seed starch, obtaining 110 g/L of glucose. However, this method involved additional operations, including starch extraction, drying, and grinding, increasing the process’s complexity [17]. Moreover, the extraction yield of starch from avocado seed is highly variable and depends on factors such as the extraction method, with values reported from 6.7% to 64% [17,33]. This variability will directly impact the overall theoretical glucose and ethanol yield of the process, highlighting the advantage of direct starch hydrolysis via microwave-assisted pretreatment over conventional multi-step approaches.

As expected, experiment 13 exhibited the highest CSF and energy consumption, reaching 1.75 and 0.182 kWh, respectively. That is, the more extreme the conditions, the higher the CSF. However, in the BBD, the major glucose concentration was achieved under less severe conditions in experiment 5, where lower CSF (1.05) and reduced energy requirement (0.078 kWh) were sufficient. Notably, the CSF obtained in experiment 5 was also lower compared to those values estimated for the pretreatment on other agroindustry biomasses that required more severe conditions, whether using microwaves, e.g., brewer’s spent grain (1.26) [7], or using conventional heating, e.g., rice straw (2.26) [34]. These findings further highlight the potential of avocado seeds to produce cost-effective fermentation products with high energy efficiency.

The pretreatment of biomass is associated with the formation of inhibitory compounds resulting from the degradation of sugars, pectin, and lignin, which produce furan aldehydes, organic acids, and phenolic compounds. These compounds can hinder or even inhibit the subsequent biochemical conversion of the pretreatment liquor into bioethanol or other bioproducts [35,36]. Therefore, the concentration of inhibitors was also analyzed, and the results are presented in Supplementary Materials Table S1. The major inhibitors detected were galacturonic acid (0.04–1.62 g/L), formic acid (0.12–0.76 g/L), and acetic acid (0.23–0.64 g/L), with the highest concentrations in the pretreatment liquors obtained within under more severe condition, and with acid, in agreement with previous findings (e.g., runs 5, 12, and 13) [7]. While the former derives from pectins [36], the latter compounds result from sugar degradation [7]. For example, glucose may be converted to HMF and formic acid, which were detected in the pretreatment liquors. In the case of HMF, concentrations of up to 0.85 g/L were found (run 13).

### 3.3. Effect of Microwave-Assisted Diluted Acid Parameters on the Studied Responses

RSM was applied to determine the significant factors that affected the studied responses, with the corresponding model equations detailed in Table 3. Quadratic equations were required to accurately describe the concentration of sugars and inhibitors, while the microwave energy consumption followed a linear model. In general, the models were statistically significant (*p*-values < 0.05). The R^2^ value for all responses was close to 1 (0.992–0.999), indicating an adequate model fit. The adjusted R^2^ values were also adequate (>0.909), indicating a strong correlation between the predicted data and experimental values. The CV data were lower than 10%, although some dispersion was observed in the case of glucose concentration. The lack-of-fit *p*-values were equal to or higher than 0.05; this suggests that the models fit the experimental data at all design points. Given their robustness, these models were selected for further validation and optimization using multiple-response optimization, as discussed in Section 3.4.

**Figure 2:**
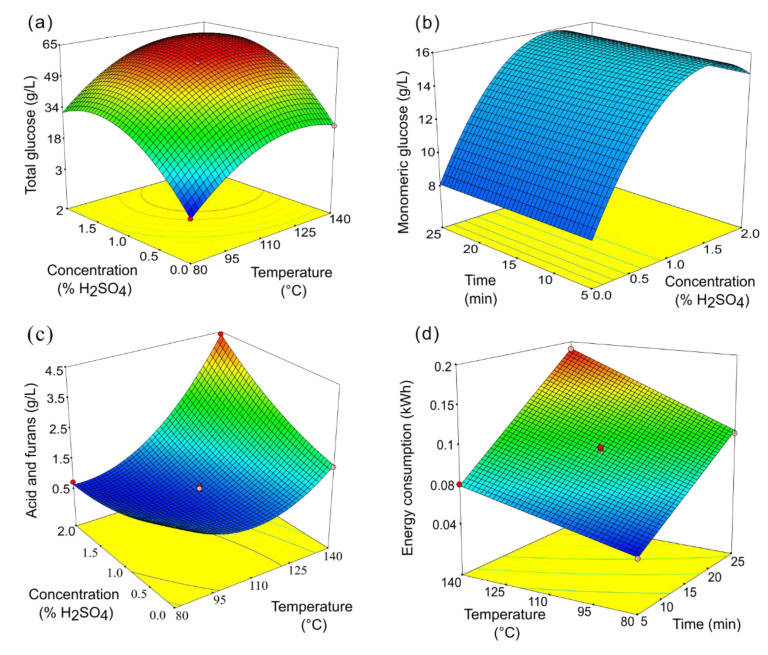
Examples of response surface responses: concentration of (**a**) total glucose, (**b**) monomeric glucose, and (**c**) acids and furans (sum of galacturonic acid, acetic acid, formic acid, levulinic acid, furfural, and hydroxymethylfurfural), and (**d**) energy consumption. In each plot, the third factor was fixed as follows (intermediate value tested in the design): Time = 15 min; temperature = 110 °C; sulfuric acid concentration: 1% *w*/*v*.

Among the tested conditions, holding temperature and sulfuric acid concentration were found to be the most critical factors affecting both sugar release and inhibitor formation (Table 3). For example, the highest values of total glucose concentration were reached from 125 °C, where almost all glucans were solubilized, aided by the acid (Figure 2a). Moreover, this factor was crucial for converting glucooligosaccharides into glucose, with the highest concentrations of glucose observed under mild acid conditions, where a maximum was reached (e.g., Figure 2b; Table 2). This can be related to the positive interaction effect between acid concentrations and temperature, favoring the degradation of sugars and resulting in the generation of organic acids and furans (Figure 2c), agreeing with a previous study [7]. Alternatively, holding time had little impact on total glucose release compared to the other factors, and its effect was not significant for glucose solubilization, as shown in Figure 2b and Table 3. This indicates that glucose in monomeric form can be obtained from avocado starch in a short time when using dilute acid in microwave pretreatment. Microwave heating offers quick and direct heating for biomass conversion, reducing extraction and pretreatment time [20,37]. It has been suggested that a synergistic effect of microwave heating and acid in hydrothermal treatments helps solubilize amorphous regions and break hydrogen bonds of polymeric sugars. This exposes more glycosidic bonds to water, enabling further hydrolysis of amorphous parts and aiding the hydrolysis of crystalline regions [37,38]. In the case of starch, it possesses amorphous regions owing to the presence of amylose, composed of linear segments of D-glucose linked by α-(1,4) bonds, while the other component, amylopectin, possesses branches attached to the main α-(1,4) chain through α-(1,6), also conferring a certain degree of crystallinity [38].

Energy consumption in microwave-assisted pretreatment varied primarily based on holding time and temperature, with holding time having a greater impact (Figure 2d; Table 3). Moreover, a negative interaction between time and the other factors also occurs for total glucose release, but is positive for total acids and furans (Table 3). This again suggests that minimizing pretreatment duration should be preferred to promote the release of free glucose and improve the quality of the pretreatment liquors for fermentation by reducing acid and furan formation. Additionally, reducing time is crucial for enhancing the energy efficiency of biorefineries employing this technology. Consequently, optimizing shorter reaction times could contribute to lower operational costs (lower energy consumption) and improved sustainability.

### 3.4. Multiple Optimization

The ideal pretreatment process should maximize the recovery of fermentable sugars while keeping the concentration of inhibitory compounds at a minimum. However, it is also important to consider the energy requirements of the process as the utilities consumption directly impacts the environmental footprint. In this context, a multiple optimization was conducted on avocado seed using the software Design-Expert to find the best combination of experimental parameters that enables maximizing the concentration of total glucose, glucose, and other fermentable sugars, while minimizing the concentration of acids and furans in the liquor and the energy consumption. The conditions predicted as those at experiment 5 (140 °C for 5 min, 1% *w*/*v* sulfuric acid), achieving a desirability of 0.86. This set of conditions was selected for replication and validation of the global model. Table 4 shows the predicted and experimental values obtained for the avocado seed with a relative error lower than 5% in all cases.

In addition, the same conditions were then applied to the entire avocado stone to compare with the seed, obtaining comparable results owing to its similar composition (Table 1). Under these conditions, about 92% of the glucose in the avocado seed and 90% in the whole stone were successfully recovered as fermentable glucose in the pretreatment liquors. These results are particularly intriguing for utilizing the avocado stone biomass, either the whole part or the seed, in a biorefinery industry when compared to other agroindustrial biomasses (e.g., brewer’s spent grain, rice straw, and olive residues). Unlike avocado seed, these residual biomasses require one or more sequential pretreatments to make cellulose accessible to enzymatic hydrolysis or additional separation steps to separate inhibitors, increasing the biorefinery steps [7,9,23,39]. Moreover, the pretreatment conditions for these biomasses seem to require higher severity, e.g., temperatures higher than 147 °C with residence times varying between 2 min (microwave technology) and 90 min (conventional heating) [7,23,34]. The results align with the work of Caballero-Sánchez and coworkers, who used diluted sulfuric acid pretreatment (~2% *w*/*v*) on avocado seed starch to produce glucose-rich hydrolysates at a lower temperature, 87 °C, but using 12 h [17]. Another study, which utilized food waste with approximately 66.3% glucans from primarily amylaceous origin, was adequately hydrolyzed using 1.5% *v*/*v* sulfuric acid (60 min at 127 °C in an autoclave) more effectively than with enzymes (α-amylase and amyloglucosidase) [40].

The presence and generation of inhibitors affect the fermentation performance, and the tolerance of microorganisms to each inhibitor varies depending on the strain. Particularly, *S. cerevisiae* is highly sensitive to acetic acid. For example, concentrations of ~5 g/L of this acid at pH 4 have been reported to delay 30 h or 40 h the lag phase of the strain Ethanol red^®^ [41]. However, the concentration of acetic acid generated under the optimized conditions was relatively low (up to 0.75 g/L). Synergism between the concentration of furfural and acetic acid in the pretreatment liquors can also affect subsequent fermentation [23], but the concentration of furfural was negligible. One possible explanation is that the severity of the pretreatment was relatively low, which limited the degradation of sugars to a high extent compared to more severe pretreatment conditions.

The chemical composition of the feedstock also affects inhibitor formation, particularly in the generation of organic acids. For example, hemicellulose degradation is highly related to the formation of organic acids and furfural [35]. In the case of avocado stone biomass, the low hemicellulosic content explains the reduced level of inhibitors observed, which is desired. Concerning galacturonic acid, which reached up to 0.5 g/L, previous research suggests that it should not affect the fermentation of glucose by *S. cerevisiae* [36].

### 3.5. Production of Bioethanol

**Figure 3:**
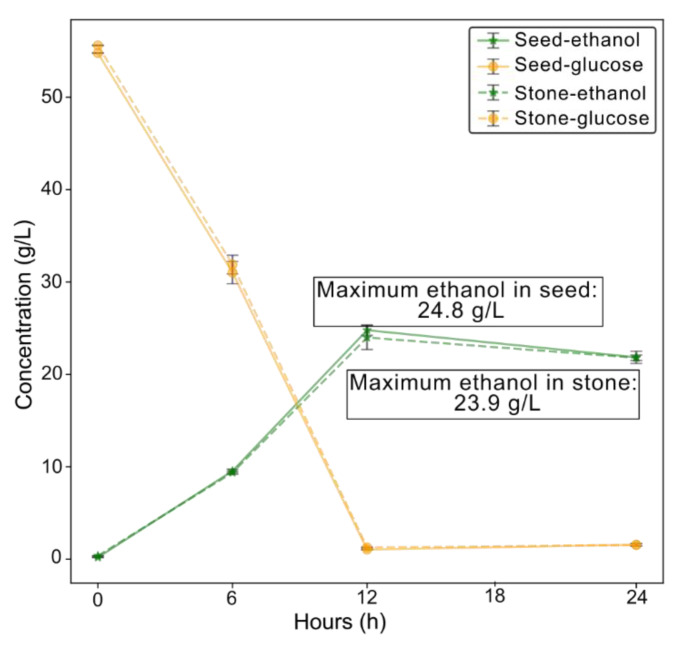
Glucose utilization (yellow) and ethanol production (green) by *Saccharomyces cerevisiae* using the glucose-rich hydrolysates obtained from avocado seed and stone.

Owing to the low concentration of inhibitory compounds in both studied pretreatment liquors, one derived from avocado seed and the other from the whole stone, they were directly subjected to fermentation under the optimized conditions without detoxification. Figure 3 shows that the maximum ethanol concentration, approximately 24 g/L, was reached at 12 h for both substrates. At this point, the microorganisms had fully consumed the available glucose. The theoretical ethanol yield obtained at this time was about 89 ± 2% and 85 ± 5% for the seed and stone, respectively. According to our findings, both avocado seeds and stones are viable feedstocks for producing bioethanol. Considering these values, it is estimated that about 24 kg of ethanol per 100 kg of avocado seed or stone can be produced under the studied solid load conditions using direct fermentation.

This proposed optimized process is also particularly advantageous because it is easy to implement, requires a short fermentation time, and does not involve the use of purification and/or enzymatic hydrolysis steps as in previous works on bioethanol production. For example, a similar theoretical yield of approximately 88% was achieved using starch from avocado stone and *S. cerevisiae* (DistilaMax^®^ DS strain) for 16 h of fermentation, but required starch extraction [17,33]. If a hydrothermal pretreatment is used, a higher temperature (e.g., 220 °C) and subsequent enzymatic hydrolysis are required to obtain a similar bioethanol potential to that of the present work (~24 kg ethanol/100 kg avocado stone) [10]. Bioethanol production from other agroindustrial wastes requires more complex processes that include detoxification to remove inhibitors with sorbents and resins, distillation or membrane separation to enhance fermentation [9,23,39].

### 3.6. Recovery of Phenolic Compounds and Characterization

Phenolic compounds from avocado are intriguing for their bioactive potential [26], and hence their content was monitored in the BBD and at optimal conditions. Under the latter conditions, the pretreatment liquor from avocado seed revealed considerable TPC, reaching 1.56 g/L (15.6 mg/g biomass), which agreed with a previous study on this biomass [42]. This represents 70–80% of the maximal concentration reached in the BBD (run 9; Supplementary Materials Table S1) or extracted by Soxhlet (Table 1), respectively. The TPC in the pretreatment liquor of avocado stone was slightly higher (1.69 g/L or 16.9 mg/g biomass) (Table 4). Therefore, as a potential strategy to implement a biorefinery process, the pretreatment liquors were extracted with ethyl acetate to separate sugars from phenolic compounds. This strategy enables the aqueous fraction rich in glucose to be directly fermented into bioethanol, while the phenolic extract (recovered by ethyl acetate) could serve as an additional bioproduct if integrated into a biorefinery cascading process. In the present case, the TPC of the extracts recovered by ethyl acetate contained hydroxycinnamic acids, which have bioactive potential as antioxidants and as anti-UV compounds in the food, pharmaceutical, and cosmetic sectors, and applications as building blocks for chemical synthesis (Table 4 and Table 5) [43].

Particularly, a total of twenty phenolic compounds were characterized by HPLC-QTOF-MS, including hydroxycinnamic acids composed of caffeoyl, coumaroyl, and feruloyl moieties linked to quinic acid, agreeing with a previous study on avocado seeds [44]. Moreover, novel compounds were detected in the extracts, including hydroxytyrosol, caffeoyl methylquinic acid, and benzaldehydes (Table 5), as they have not been reported before in avocado stone [26,27,28]. The former, hydroxytyrosol, is another highly valuable phenolic compound with marketable potential. This compound has previously been linked to glucose in avocado seed (cv. Hass) extracted with 80% methanol [26,27,28] and fermented with lactic acid bacteria [45]. The latter compound might be susceptible to acid hydrolysis, which could account for its presence in the extract as a free form. Methods like acid hydrolysis [46], microbial fermentation [45], and the direct use of enzymes [47] can (bio)transform phenolic compounds, for example, hydroxytyrosol glycosidic derivatives into hydroxytyrosol. Other research found that dihydroxybenzoic acid and other simple phenolic compounds can be derived from the breakdown of flavanols, as it was observed after fermentation of avocado seeds with *Aspergillus* spp. [48]. Moreover, the phenolic profile was different from that of an ethyl acetate extract from Hass avocado seed obtained after being pretreated with water at 220 °C and microwaves [10]. These authors found the phenolic aldehyde vanillin as the main compound, along with ethylvainillin, some phenolic acids (phthalic acid, ferulic acid, salicylic acid, and *p*-coumaric acid), and two flavonoids (luteolin and quercetin). This suggests that the production process of avocado phenolic compounds might impact the phenolic profile, along with other factors like the origin and cultivar [13]. Among these compounds, hydroxycinnamic acids like 5-caffeoylquinic acid (or chlorogenic acid) have been largely studied for food, pharmaceutical, and cosmetic applications as they present beneficial biological activities, including antioxidant and antimicrobial activity [49]. Chlorogenic acid and its isomer, 1-caffeoylquinic acid, highly contributed to the antioxidant activity of avocado peel extracts [50], and the former component had a crucial role in the antibacterial activity of this type of extract [51]. Hydroxytyrosol has been linked to antioxidant effects in vivo and in food systems, along with other relevant bioactive properties (anti-inflammatory, anti-cancer, skin protection, etc.) [52,53]. Hydroxybenzoid acids, particularly salicylic acid (2-hydroxybenzoic acid), have significant relevance in cosmetic and medical formulations and for plant protection [54].

Some studies have revealed that the cyanogenic glycoside amygdalin and the acetogenin persin occur in avocado seeds at trace (ng/g) and low levels (<2 mg/g), respectively [55]. Amygdalin, after excessive ingestion of apricot seeds, resulted in some adverse effects, although the content of amygdalin in this type of seed is much higher (0.16–79 mg/g) than in avocado seed [56]. Persin can be toxic at high doses of consumption for some animals, like sheep, goat, and horses [57]. Under the analytical conditions used, neither of these compounds was detected in the studied extracts by looking at their *m/z* values of monoisotopic ions in the negative (456.1511 for amygdalin and 379.2854 for persin) and positive ionization modes (458.1657 for amygdalin and 381.2999 for persin). These compounds are prone to degradation under acidic conditions and heat treatments [55,56].

Overall, these findings highlight the relevance of the phenolic compounds recovered for future applications.

### 3.7. Characterization of the Pretreated Solids

The pretreated solids obtained after the microwave-diluted acid pretreatment of the avocado seed and stone were preliminarily characterized to assess their potential for further valorization (Table 6). As expected, lignin was the main component, with values of 67.02% and 74.18% *w*/*w* for the seed and the stone-derived solids, respectively. This slight difference could be related to the presence of lignin derived from the seed coat in the stone, which contained more lignin (Table 1). The solids also contained residual nitrogenous material and glucans as impurities (Table 6). It is also worth noting that the lignin value may be overestimated due to the presence of waxes, as nearly half of the extractable compounds are insoluble in water (Table 1).

Compared to the raw biomasses, the carbon content in both pretreated solids increased from about 44–45% (Table 1) to 56%, enhancing the HHV, i.e., the amount of heat released during complete combustion. The HHV of both lignin-rich solids was around 22-23 kJ/g, falling in the range of purified lignins obtained from other agroindustrial and lignocellulosic wastes (e.g., 17.3–29.2 kJ/g), thereby indicating potential for thermochemical conversion [58,59].

**Figure 4:**
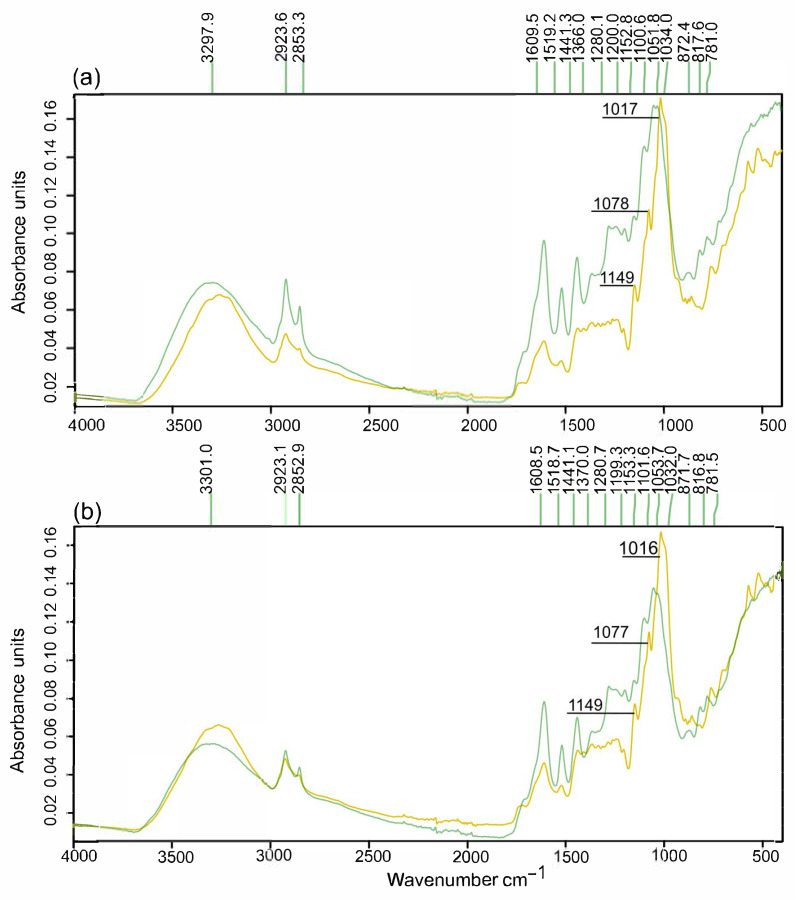
FTIR spectra of (**a**) avocado seed (yellow) and pretreated solid (green), and (**b**) avocado stone (yellow) and pretreated solid (green).

The FTIR results aligned with the findings mentioned above. This analysis revealed that the bands at about 1017 cm^−1^ and 1078 cm^−1^—related to starch [60]—were present in the avocado seed and stone and disappeared after the pretreatment (Figure 4a,b). The band at about 1149 cm^−1^ could be related to C-O-C stretching in sugar polymers, according to previous studies [60,61]. The persistence of this peak, albeit with reduced intensity in the pretreated solids (Figure 4a,b), suggests that non-hydrolyzed cellulose/glucans remained, which aligns with chemical composition data (Table 6).

Alternatively, the bands related to lignin were enhanced in both pretreated solids; for example, 2924 cm^−1^ (CH_2_ asymmetric vibration of guaiacyl and syringyl units of lingin), about 1610 cm^−1^ (vibration of C=C aromatic rings), 1519 cm^−1^ (aromatic skeletal vibration coupled with C=O stretching), 1280 cm^−1^ (C–O and glucopyranose cycle, guaiacyl symmetric vibration), and 1034 cm^−1^ (C–H in-plane deformation in guaiacyl/primary alcohol C–O deformation) [61]. The wavelength at about 1440 cm^−1^ is also associated with both aromatic skeleton vibration and -CH deformation [62].

Dilute acid pretreatment favors hemicellulose and starch hydrolysis in biomass [63,64]. It has been found that most cellulose, owing to its higher crystallinity compared to these polymeric carbohydrates, and lignin, with its stable C–C bonds, remain in the resulting pretreated solids [63,65,66]. Nonetheless, the rate of removal of all these components, including lignin, depends on both the type of biomass and the conditions applied in dilute acid pretreatment, such as acid concentration, temperature, and time. For example, lignin removal may vary from negligible lignin removal using sweet sorghum leaves to 41% with cotton stalk [63,65,66]. Therefore, the optimal conditions applied using microwaves appear to favor the hydrolysis of starch to lignin in avocado stone biomass, but more severe conditions could change the results. It can be explained as starch from the Hass avocado seeds presents a low degree of crystallinity owing to its high content of amylose, up to 39.6%, and its low content of short amylopectin chains that may favor its hydrolysis [67,68].

### 3.8. Perspectives of an Avocado Stone Biorefinery

The use of avocado stone or seed to formulate new foods is growing [40,55]. However, the levels of orally toxic compounds like persin and amygdalin should be monitored in the final food/feed products to ensure they are not higher than in the avocado pulp, since no human studies have been conducted on persin’s toxicity [55]. The presence of these types of compounds can also limit the use of avocado waste as feed at high doses [55,57]. As an alternative application beyond food, the present study demonstrates that the entire avocado stone, including the seed, can serve as feedstock for 2G biorefineries to obtain biofuels along with phenolic compounds, promoting food sustainability. For industrial use, its direct application eliminates the need to separate the coat from the seed, thereby reducing the number of operational steps and achieving similar yields to those of the seed. Using the avocado stone, the biorefinery process could potentially produce 24 kg of bioethanol per 100 kg, along with 0.25 kg of phenolic compounds and 37.6 kg of lignin-rich solid (~22 kJ/g) for biofuel use (Supplementary Materials Figure S2).

For effective and reliable scaling considering these data, it is crucial to clearly define the processing scale of the biorefinery—whether small or large—and consider regional production, such as the quantity of waste from the avocado processing industry and unmarketable avocados [31]. As an example, to extrapolate our findings in a real-world scenario, one of the leading guacamole producers in Spain generates about 593 t/yr of dry avocado stone for every 6000 t/yr of guacamole produced. Based on the mass balance in Supplementary Materials Figure S2, about 180.4 m^3^ of bioethanol could be generated annually. Under current Spanish regulations, this volume of ethanol would enable the production of 1804 m^3^ of E10 fuel, which is equivalent to a transportation displacement of approximately 17.9 million km/yr. Beyond bioethanol production, this biorefinery proposal would have economic, social, and environmental advantages. For example, adopting biofuels, such as bioethanol, could reduce transportation costs for companies while contributing to decarbonization. Additionally, the process could yield 1.5 t of phenolic compounds (purified extract), including marketable hydroxycinnamic acids for food, nutraceutical, and cosmeceutical applications, as well as 223 t of lignin-rich solid, which can be used as a biofuel for heating in the avocado industry to achieve self-sustainability or in nearby facilities. In Mexico, the leading avocado producer, biorefineries based on stone could supply over 13 times the aforementioned amounts, considering that guacamole exports could reach approximately 80,000 tons per year [69].

Another aspect to consider for future scaling is that although microwave technology for biorefining is not yet mature, it offers an energy-efficient method that can lower operational costs during pretreatment owing to its reduced hydrolysis time. This can counteract the investment cost compared to conventional heating, which is less energy-efficient. Bottlenecks in the technology involve the design and control of microwave reactors at an industrial scale to improve microwave penetration and heat distribution for large volumes [37]. Moreover, the application of dilute sulfuric acid has shown a faster production of reducing sugars and better ethanol yield than the use of enzymes on amylaceous food waste [70]. All of this appears promising for the future scaling of avocado conversion by microwave-assisted dilute acid conversion. Additional techno-economic and environmental analyses are needed to compare the current approach with using conventional heating for dilute acid pretreatment or the use of enzymes.

## 4. Conclusions

The optimized diluted acid pretreatment using microwaves effectively enabled the recovery of glucose in the liquor from avocado stone biomass under low severity, minimizing the production of inhibitors. It achieved more than 90% glucan solubilization, which was shortly fermented by *S. cerevisiae*, without detoxification, yielding more than 85% ethanol. The pretreatment fractionation yielded a lignin-rich solid with an HHV of approximately 22 kJ/g, suitable for thermochemical applications. Moreover, it is possible to recover phenolic extracts rich in marketable hydroxycinnamic acids and with hydroxytyrosol from the pretreatment liquor. These compounds have significant potential for use in food preservation, functional ingredients, and cosmetic products. Overall, this integrated biorefinery approach provides a sustainable and innovative strategy for the full valorization of the studied avocado waste, with the potential to contribute to achieving circular economy goals and promoting industrial self-sufficiency. Future studies should focus on scaling and conducting a techno-economic and life cycle assessment of the biorefinery proposal. This can be useful for comparing the approach with conventional ones that use dilute acids and enzymes. Moreover, the purification of phenolic compounds from the pretreatment liquor can be tested using different strategies to increase the yield and potential income.

## Figures and Tables

**Table 1:** Chemical characterization of raw and extractives of the seed coat, seed, and stone of avocado. Values are expressed as a percentage of dry weight, except moisture, which is expressed on a fresh basis.

Component	Seed Coat	Seed	Stone
Compositional analysis			
Moisture (%)	48.1 ± 2.9	61.5 ± 2.5	60.6 ± 2.3
Aqueous extractives (%)	28.73 ± 3.45	17.17 ± 3.29	17.49 ± 0.90
Ethanolic extractives (%)	4.64 ± 0.83	11.33 ± 1.96	10.15 ± 0.18
Hexanic extractives (%)	9.09 ± 1.09	4.73 ± 0.09	4.64 ± 0.23
Glucans ^1^ (%)	11.60 ± 3.24	52.17 ± 5.29	53.30 ± 0.54
Starch (%)	0.48 ± 0.06	42.78 ± 0.29	41.66 ± 2.09
Hemicellulose (%) ^2^	3.97 ± 1.02	1.02 ± 0.28	1.27 ± 0.10
Xylose (%)	1.35 ± 0.44	n.d.	n.d.
Galactose (%)	1.21 ± 0.15	0.99 ± 0.25	1.14 ± 0.08
Arabinose (%)	1.83 ± 0.52	0.13 ± 0.06	0.26 ± 0.03
Mannose (%)	0.06 ± 0.04	n.d.	n.d.
Galacturonic acid (%)	0.57 ± 0.06	0.40 ± 0.02	0.58 ± 0.03
Total lignin (%) ^3^	28.33 ± 1.50	13.08 ± 1.17	14.93 ± 2.75
Acid-soluble lignin (%)	0.83 ± 0.24	0.77 ± 0.07	0.675 ± 0.001
Acid insoluble lignin (%)	27.50 ± 1.26	12.32 ± 1.10	14.25 ± 2.75
Protein ^4^ (%)	6.15 ± 0.12	3.28 ± 0.12	2.93 ± 0.08
Ash (%)	4.34 ± 0.36	2.63 ± 0.01	2.62 ± 0.01
Acetyl group (%)	0.32 ± 0.04	n.d.	n.d.
Composition of extractives			
Glucose ^5^ (%)	1.14 ± 0.02	3.08 ± 0.55	3.24 ± 0.34
Xylose ^5^ (%)	0.68 ± 0.06	0.06 ± 0.03	0.13 ± 0.01
Galactose ^5^ (%)	0.97 ± 0.13	0.22 ± 0.03	0.21 ± 0.03
Arabinose ^5^ (%)	2.12 ± 0.08	0.35 ± 0.08	0.30 ± 0.05
Manose ^5^ (%)	0.26 ± 0.05	0.079 ± 0.002	0.08 ± 0.01
Galacturonic acid ^5^ (%)	0.92 ± 0.04	0.38 ± 0.06	0.33 ± 0.08
Acetic acid (%)	1.56 ± 0.31	0.31 ± 0.03	0.31 ± 0.04
TPC (%)	7.42 ± 0.29	2.00 ± 0.02	1.99 ± 0.32
Ultimate analysis			
C (%)	46.00 ± 0.36	43.81 ± 0.19	44.73 ± 0.45
H (%)	5.46 ± 0.16	6.03 ± 0.13	6.14 ± 0.12
N (%)	0.98 ± 0.02	0.53 ± 0.02	0.47 ± 0.01
S (%)	0.07 ± 0.02	0.05 ± 0.01	0.08 ± 0.01
HHV ^6^ (kJ/g)	18.44	17.49	17.89
HHV ^7^ (kJ/g)	ND	16.90 ± 0.09	16.53 ± 0.29

Abbreviations: C, carbon; H, hydrogen; HHV, high heating value; N, nitrogen; n.d., non-detected; ND, not determined; S, sulfur; TPC, total phenolic content. ^1^ as glucose, including starch and cellulose. ^2^ Calculated as the sum of hemicellulosic carbohydrates (xylose, galactose, arabinose, and mannose). ^3^ Calculated by summing acid-soluble and insoluble lignin. ^4^ Calculated as 6.25 × N. ^5^ Calculated as the sum of monomeric, dimeric, and oligomeric carbohydrates/derivatives. ^6^ HHV estimated using Equation (1) (Section 2.3). ^7^ HHV estimated with a bomb calorimeter (Section 2.3).

**Table 2:** Box–Behnken experimental design for the microwave-diluted acid pretreatment of the avocado seed, concentration of total glucose (glucose and glucooligosaccharides), glucose, and other sugars, glucose recovery, energy consumed, and combined severity factor.

Run	Temperature (°C)	Time (min)	H_2_SO_4_ (% *w*/*v*)	Total Gluc. ^1^ (g/L)	Gluc. (g/L)	Other Sugars ^2^ (g/L)	Gluc. Rec. ^3^ (%)	Energy (kWh) ^4^	CSF ^5^
1	110 (0)	15 (0)	1 (0)	58.75	12.46	13.07	96.0	0.106	0.64
2	110 (0)	15 (0)	1 (0)	57.70	12.96	12.93	94.4	0.104	0.64
3	110 (0)	25 (1)	2 (1)	56.66	48.58	13.24	92.9	0.155	1.01
4	110 (0)	5 (−1)	2 (1)	58.93	13.99	13.08	96.4	0.060	0.31
5	140 (1)	5 (−1)	1 (0)	59.48	59.48	14.40	97.5	0.078	1.05
6	80 (−1)	15 (0)	2 (1)	42.76	3.34	10.77	69.9	0.081	−0.09
7	110 (0)	15 (0)	1 (0)	57.46	12.04	12.62	94.1	0.105	0.64
8	80 (−1)	15 (0)	0 (−1)	3.26	3.10	10.52	5.3	0.078	ND
9	140 (1)	15 (0)	0 (−1)	27.07	3.19	14.17	44.3	0.125	ND
10	80 (−1)	15 (0)	1 (0)	38.14	3.07	11.48	62.4	0.113	−0.02
11	110 (0)	15 (0)	1 (0)	58.30	12.81	12.88	95.5	0.106	0.64
12	140 (1)	15 (0)	2 (1)	57.06	57.06	12.89	93.3	0.132	1.67
13	140 (1)	25 (1)	1 (0)	58.48	58.48	13.36	95.7	0.182	1.75
14	110 (0)	15 (0)	1 (0)	57.29	12.78	13.19	93.7	0.101	0.64
15	80 (−1)	5 (−1)	1 (0)	30.95	3.07	12.74	50.6	0.040	−0.72
16	110 (0)	25 (1)	0 (−1)	34.50	3.03	15.19	56.5	0.149	ND
17	110 (0)	5 (−1)	0 (−1)	40.16	3.13	15.32	65.8	0.060	ND

The coded terms of the factors studied (−1, 0, 1) are shown in parentheses in columns 2, 3, and 4. Abbreviations: CSF, combined severity factor; Gluc., glucose; Gluc. rec., glucose recovery. ^1^ Total glucose refers to all glucose released after a second hydrolysis step (i.e., glucose and glucooligosaccharides). ^2^ Other sugars refers to arabinose plus those sugars co-eluting with xylose. ^3^ Gluc. rec. was estimated as total glucose (glucose plus glucooligosaccharides) in liquors ×100/maximum glucose that could be theoretically released. ^4^ Energy measured for the ramp + holding time. ^5^ ND stands for ‘not determined,’ as the CSF is only applicable for acid-catalyzed pretreatments.

**Table 3:** Models obtained by the Response Surface Methodology for the responses studied in the Box–Behnken Design of the microwave-dilute acid pretreatment of avocado seed. Only significant coefficients at *p* < 0.1 are considered.

Parameter ^1^	Total Glucose ^2^ (g/L)	Glucose (g/L)	Other Sugars (g/L)	Acids and Furans ^3^ (g/L)	TPC (g/L)	Energy Consumption (kW h)
Equation terms						
$\beta_{0}$	57.90	12.09	12.94	0.86	1.39	0.104
A	1.79	-	−0.28	0.10	−0.01	0.045
B	12.19	26.79	0.90	0.99	0.08	0.026
C	14.49	3.13	−0.91	0.32	−0.13	0.002
AB	−2.05	-	-	0.84	−0.05	0.008
AC	−3.17	-	-	0.10	−0.25	-
BC	-	-	-	0.89	−0.60	-
A ^2^	1.26	-	0.83	−0.35	−0.32	-
B ^2^	−12.40	19.58	−0.77	1.04	−0.08	-
C ^2^	−15.61	−3.48	0.45	0.31	0.13	-
Statistics						
Model (*p*-value)	<0.0001	<0.0001	<0.0001	<0.0001	<0.0001	<0.0001
Lack-of-fit	0.30	0.05	0.10	0.05	0.50	0.40
CV (%)	1.4	12.6	2.67	6.3	3.6	2.2
R^2^	0.999	0.992	0.946	0.998	0.994	0.997
Adjusted R^2^	0.998	0.989	0.909	0.994	0.986	0.996

-, not estimated coefficient value as the factors were not significant. Abbreviations: CV, coefficient of variation; TPC, total phenolic content. ^1^ A, holding time (min); B, holding temperature (°C); C, sulfuric acid concentration (% *w*/*v*). ^2^ Values after a second hydrolysis step (glucose + glucooligosaccharides). ^3^ Sum of galacturonic acid, acetic acid, formic acid, levulinic acid, furfural, and hydroxymethylfurfural.

**Table 4:** Predicted and experimental values obtained at optimal pretreatment conditions (140 °C for 5 min, 1% *w*/*v* sulfuric acid) for avocado seed and replication on avocado stone.

Component	Predicted Value	Experimental Value	Relative Error (%) ^4^	Experimental Value
	Seed			Stone
Total glucose (g/L) ^1^	58.67	56.80 ± 0.01	3.2	55.95 ± 0.08
Glucose (g/L)	58.36	56.60 ± 0.33	3.0	55.46 ± 0.08
Other carbohydrates (g/L)	14.21	13.92 ± 0.04	2.0	15.05 ± 0.59
Acids and furans (g/L) ^2^	1.56	1.53 ± 0.04	1.6	1.813 ± 0.009
Galacturonic acid (g/L)	-	0.43 ± 0.15	-	0.493 ± 0.003
Formic acid (g/L)	-	0.25 ± 0.01	-	0.219 ±0.003
Acetic acid (g/L)	-	0.48 ± 0.01	-	0.75 ± 0.02
Levulinic acid (g/L)	-	0.032 ± 0.003	-	0.034 ± 0.003
Furfural (g/L)	-	n.d.	-	n.d.
HMF (g/L)	-	0.34 ± 0.02	-	0.30 ± 0.02
TPC (g/L)	-	1.56 ± 0.07	-	1.69 ± 0.08
TPC (g/L) in extracts ^3^	-	0.24 ± 0.03	-	0.25 ± 0.02
Energy consumption (kWh)	0.077	0.079 ± 0.000	2.5	0.080 ± 0.000

Abbreviations: n.d., not detected; TPC, total phenolic content; HMF, hydroxymethylfurfural. ^1^ Values after a second hydrolysis step. ^2^ Sum of galacturonic acid, acetic acid, formic acid, levulinic acid, furfural, and HMF, which were optimized together. ^3^ Extracts obtained by liquid–liquid extraction with ethyl acetate. ^4^ Estimated as (|Experimental Value − Theoretical Value|/|Theoretical Value|) × 100.

**Table 5:** Phenolic compounds characterized in the ethyl acetate extracts and obtained from avocado seed and stone by liquid chromatography-mass spectrometry.

RT (min)	Exp. *m*/*z*	Molecular Formula	Score	Error (ppm)	Main MS/MS Fragments	Proposed Compound
Negative ionization mode						
**1.22**	**167.04**	**C_8_H_8_O_4_**	**98**	**0.03**	**123.04, 108.02, 109.03, 93.03**	**Dihydroxyphenylacetic acid**
1.37	153.06	C_8_H_10_O_3_	99	−0.49	123.04	Hydroxytyrosol
**1.57**	**153.02**	**C_7_H_6_O_4_**	**99**	**0.08**	**109.03, 108.02, 91.02**	**Dihydroxybenzoic acid**
**2.32**	**137.02**	**C_7_H_6_O_3_**	**99**	**−0.91**	**119.01, 108.02, 92.03**	**Hydroxybenzoic acid**
**2.37**	**353.09**	**C_16_H_18_O_9_**	**96**	**−1.37**	**191.06, 179.03, 135.04**	**Caffeoylquinic acid**
2.73	177.02	C_9_H_6_O_4_	99	0.21	149.02, 121.03, 93.04	Unknown
3.30	337.09	C_16_H_18_O_8_	99	−1.80	191.06, 163.04, 119.05	Coumaroylquinic acid
3.93	337.09	C_16_H_18_O_8_	97	−0.45	191.05, 163.04, 119.05	Coumaroylquinic acid is. 1
**5.29**	**353.09**	**C_16_H_18_O_9_**	**99**	**−0.75**	**191.06, 179.06, 135.04**	**Caffeoylquinic acid is. 1**
5.44	337.09	C_16_H_18_O_8_	99	−0.53	173.05	Coumaroylquinic acid is. 2
5.02	367.10	C_17_H_20_O_9_	98	0.46	193.05, 134.04	Ferulolylquinic acid
5.82	367.10	C_17_H_20_O_9_	98	0.01	161.02	Caffeoyl-methylquinic acid
6.53	337.09	C_16_H_18_O_8_	98	1.09	173.05	Coumaroylquinic acid is. 3
7.31	367.10	C_17_H_20_O_9_	95	1.90	193.05, 173.04	Ferulolylquinic acid is. 1
7.31	367.10	C_17_H_20_O_9_	96	−0.22	-	Ferulolylquinic acid is. 2
15.66	387.14	C_21_H_24_O_7_	100	−0.14	187.11	Unknown
Positive ionization mode						
**0.69**	**139.04**	**C_7_H_6_O_3_**	**99**	**−0.70**	**110.04, 81.03, 55.06, 53.04**	**Dihydroxybenzaldehyde**
**0.97**	**127.04**	**C_6_H_6_O_3_**	**99**	**1.79**	**109.03, 81.03, 55.06, 53.04**	**Pyrogallol**
2.30	139.04	C_7_H_6_O_3_	99	1.07	110.04, 93.03, 65.04	Hydroxybenzoic acid
4.88	169.05	C_8_H_8_O_4_	99	−1.35	109.03, 81.03, 53.04	Dihydroxy-methoxy benzaldehyde
5.94	139.04	C_7_H_6_O_3_	97	3.10	55.06, 53.04	Dihydroxybenzaldehyde is. 1

Abbreviations: -, not fragmented; Exp., experimental; is., isomer; MS, mass spectrometry; RT, retention time. The main compounds are highlighted in bold according to their peak intensity.

**Table 6:** Chemical characterization on a dry basis of pretreated solids from avocado seed and stone.

Component	Seed	Stone
Compositional analysis		
Glucans ^1^ (%)	10.96 ± 1.47	12.98 ± 0.29
Glucose (%)	12.06 ± 0.51	14.29 ± 0.32
Hemicellulose ^2^ (%)	n.d.	n.d.
Galacturonic acid (%)	0.23 ± 0.02	0.27 ± 0.01
		
Total lignin ^3^ (%)	67.02 ± 2.39	74.18 ± 0.49
Acid-soluble lignin (%)	1.51 ± 0.16	1.66 ± 0.10
Acid-insoluble lignin (%)	65.51 ± 2.24	72.51 ± 0.39
Protein ^4^ (%)	10.56 ± 0.02	8.73 ± 0.03
Ash (%)	0.315 ± 0.004	0.269 ± 0.007
Ultimate analysis		
C (%)	55.76 ± 0.54	56.36 ± 0.07
H (%)	6.64 ± 0.11	6.41 ± 0.08
N (%)	1.69 ± 0.01	1.40 ± 0.01
S (%)	0.16 ± 0.02	0.15 ± 0.03
HHV ^5^ (kJ/g)	22.71	22.97
HHV ^6^ (kJ/g)	22.84 ± 0.03	22.35 ± 0.14

Abbreviations: C, carbon; H, hydrogen; HHV, high heating value; N, nitrogen; n.d., non-detected; S, sulfur. ^1^ Calculated as glucose. ^2^ Calculated as the sum of hemicellulosic sugars. ^3^ Calculated by summing acid-soluble and insoluble lignin. ^4^ Calculated as 6.25 × N. ^5^ HHV estimated with Equation (1) (Section 2.3). ^6^ Calculated with a bomb calorimeter (Section 2.3).

## Data Availability

The data are contained within the article or Supplementary Materials.

## Supplementary Materials

The following supporting information can be downloaded at: https://www.mdpi.com/article/10.3390/foods14183160/s1, Figure S1: Proportions of avocado and stone parts in fresh weight (f.w.); Figure S2: Summarized mass balance of the biorefinery processing of avocado stone; Table S1: Fermentation inhibitors produced in the pretreatment liquors by microwave-assisted acid pretreatment.

## Abbreviations

The following abbreviations are used in this manuscript:

ATR	Attenuated total reflectance
BBD	Box–Behnken design
CSF	Combined severity factor
FTIR	Fourier transform infrared
GAE	Gallic acid equivalents
HHV	High heating value
HMF	Hydroxymethylfurfural
HPLC	High-performance liquid chromatography
MS	Mass spectrometry
RSM	Response surface methodology
RT	Retention time
TPC	Total phenolic content
