# Supplementary material for: Sustainable Bioethanol Production and Phenolic Compounds from Avocado Stone Biomass Based on Microwave Pretreatment
Source: Foods. 2025 Sep 10;14(18):3160. doi: 10.3390/foods14183160 (PMC12468688; doi:10.3390/foods14183160)
Supplement: Supplementary file 1 [file foods-14-03160-s001.zip › foods-3807379-supplementary.pdf]

Sustainable Production from Avocado Stone Biomass Bioethanol and Phenolic Compounds Based on Microwave Pretreatment

Luis Carlos Morán-Alarcón [1,2], María del Mar Contreras [1,2,*], Juan Miguel Romero-García[1,2], Ángel Galán-Martín[1,2], Eulogio Castro[1,2]

[1] Department of Chemical, Environmental and Materials Engineering, Universidad de Jaén, Campus Las Lagunillas, Jaén, 23071, Spain; lmoran@ujaen.es, jrgarcia@ujaen.es, galan@ujaen.es, ecastro@ujaen.es
[2] Institute of Biorefineries Research (I3B), University of Jaén, 23071 Jaén, Spain
* Correspondence: mcgamez@ujaen.es

Supplementary material

Table S1. Fermentation inhibitors produced in the pretreatment liquors by microwave-assisted acid pretreatment.

Run	Galacturonic acid (g/L)	Formic acid (g/L)	Acetic acid (g/L)	Levulinic acid (g/L)	HMF (g/L)	Furfural (g/L)	TPC (g/L)
1	0.19	0.12	0.47	n.d.	0.03	n.d.	1.41
2	0.23	0.18	0.43	n.d.	0.03	n.d.	1.45
3	0.41	0.19	0.54	0.01	0.11	n.d.	0.81
4	0.26	0.21	0.47	n.d.	0.01	n.d.	1.31
5	0.42	0.30	0.51	0.02	0.34	n.d.	1.34
6	0.16	0.20	0.35	n.d.	n.d.	n.d.	1.01
7	0.21	0.18	0.42	n.d.	0.02	n.d.	1.34
8	0.60	0.21	0.23	n.d.	n.d.	n.d.	0.87
9	1.13	0.37	0.40	n.d.	n.d.	n.d.	2.25
10	0.67	0.20	0.32	n.d.	n.d.	n.d.	0.95
11	0.22	0.17	0.44	n.d.	0.02	n.d.	1.35
12	1.62	0.76	0.64	0.51	0.79	0.08	0.83
13	1.20	0.59	0.58	0.26	0.85	0.07	0.98
14	0.24	0.20	0.45	n.d.	0.02	n.d.	1.39
15	0.38	0.22	0.27	n.d.	n.d.	n.d.	0.89
16	0.04	0.20	0.25	n.d.	n.d.	n.d.	1.58
17	0.07	0.22	0.28	n.d.	n.d.	n.d.	1.10

Abbreviations: HMF, hydroxymethylfurfural; n.d., not detected; TPC, total phenolic content.

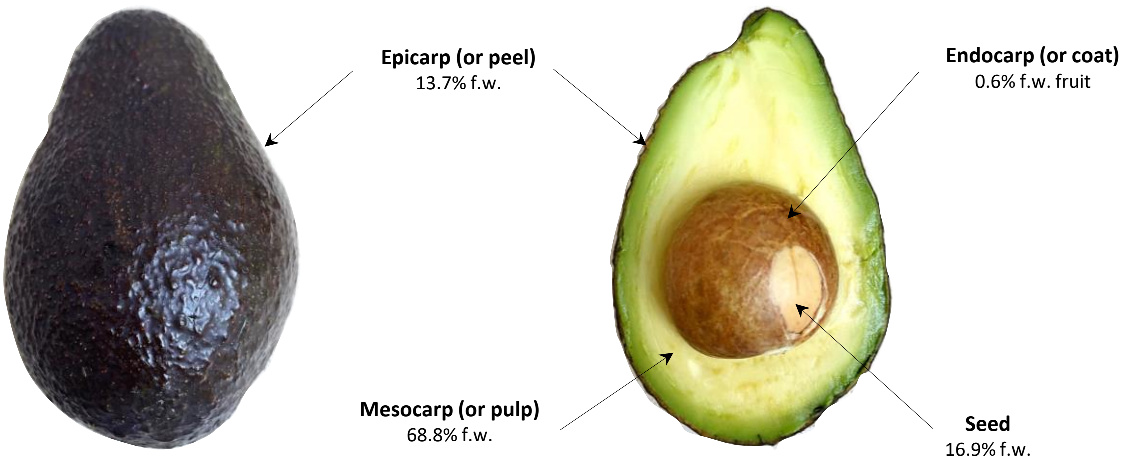

Figure S1. Proportions of avocado and stone parts in fresh weight (f.w.).

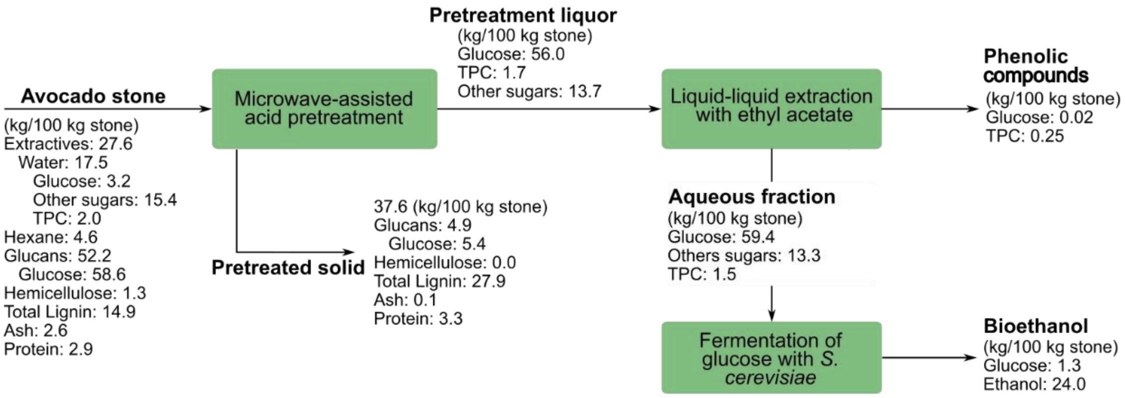

Figure S2. Summarized mass balance of the biorefinery processing of avocado stone.
